# Anti-PTK7 Monoclonal Antibodies Inhibit Angiogenesis by Suppressing PTK7 Function

**DOI:** 10.3390/cancers14184463

**Published:** 2022-09-14

**Authors:** Si Won Oh, Won-Sik Shin, Seung-Taek Lee

**Affiliations:** Department of Biochemistry, College of Life Science and Biotechnology, Yonsei University, Seoul 03722, Korea

**Keywords:** PTK7, receptor protein tyrosine kinase, angiogenesis, neutralizing antibody, KDR

## Abstract

**Simple Summary:**

PTK7 is a catalytically defective receptor protein tyrosine kinase. We previously demonstrated that PTK7 enhances angiogenesis by interacting with KDR, a vascular endothelial growth factor (VEGF) receptor important for angiogenesis, and activating it through oligomerization. To control angiogenesis by inhibiting PTK7 function, we developed anti-PTK7 monoclonal antibodies (mAbs). The selected PTK7 mAbs reduced VEGF-induced angiogenic phenotypes of endothelial cells and angiogenesis ex vivo and in vivo. The PTK7 mAbs also inhibited VEGF-induced KDR activation in endothelial cells and its downstream signaling and PTK7–KDR interaction. Our results show that the PTK7 mAbs inhibit angiogenesis by blocking PTK7 function. Therefore, PTK7 mAbs could be applied as therapeutics to control angiogenesis-associated diseases such as metastatic cancers.

**Abstract:**

PTK7, a catalytically defective receptor protein tyrosine kinase, promotes angiogenesis by activating KDR through direct interaction and induction of KDR oligomerization. This study developed anti-PTK7 monoclonal antibodies (mAbs) to regulate angiogenesis by inhibiting PTK7 function. The effect of anti-PTK7 mAbs on vascular endothelial growth factor (VEGF)-induced angiogenic phenotypes in human umbilical vascular endothelial cells (HUVECs) was examined. Analysis of mAb binding with PTK7 deletion mutants revealed that mAb-43 and mAb-52 recognize immunoglobulin (Ig) domain 2 of PTK7, whereas mAb-32 and mAb-50 recognize Ig domains 6–7. Anti-PTK7 mAbs inhibited VEGF-induced adhesion and wound healing in HUVECs. mAb-32, mAb-43, and mAb-52 dose-dependently mitigated VEGF-induced migration and invasion in HUVECs without exerting cytotoxic effects. Additionally, mAb-32, mAb-43, and mAb-52 inhibited capillary-like tube formation in HUVECs, and mAb-32 and mAb-43 suppressed angiogenesis ex vivo (aortic ring assay) and in vivo (Matrigel plug assay). Furthermore, mAb-32 and mAb-43 downregulated VEGF-induced KDR activation and downstream signaling and inhibited PTK7–KDR interaction in PTK7-overexpressing and KDR-overexpressing HEK293 cells. Thus, anti-PTK7 mAbs inhibit angiogenic phenotypes by blocking PTK7–KDR interaction. These findings indicate that anti-PTK7 mAbs that neutralize PTK7 function can alleviate impaired angiogenesis-associated pathological conditions, such as cancer metastasis.

## 1. Introduction

Receptor protein tyrosine kinases (RPTKs), which are active tyrosine kinases, play an important role in signal transduction involved in cellular processes, such as proliferation, differentiation, and metabolism [1]. Of the 58 human RPTKs, the following eight are pseudokinases that do not exhibit tyrosinase kinase catalytic activity: PTK7, ERBB3, EPHB6, EPHA10, STYK1, ROR1, ROR2, and RYK [2].

PTK7 comprises an extracellular region with seven immunoglobulin (Ig)-like loops, a transmembrane domain, and a cytosolic region containing a defective tyrosine kinase catalytic domain [3]. Previous studies have reported that PTK7 functions as a co-receptor in the semaphorin/plexin signaling pathway [4], as well as in planar cell polarity and non-canonical Wnt signaling pathways [5,6,7,8,9] during developmental processes. Additionally, PTK7 is associated with tumorigenesis. The expression of PTK7, which is upregulated in various malignancies, is negatively correlated with disease-free survival and/or overall survival in patients with cancer [10,11,12,13,14,15,16,17,18,19]. PTK7 potentiates oncogenic signaling by functioning as a co-receptor for active RPTKs, such as FGFR1 [20]. The expression of PTK7 is also upregulated in endothelial cells, especially during tube formation [21].

Angiogenesis, which is the process of formation of new blood vessels, involves protease production for extracellular matrix degradation, chemotactic migration and proliferation of endothelial cells, and capillary-like tube formation induced by angiogenic factors, such as vascular endothelial growth factor (VEGF) and basic fibroblast growth factor (bFGF; also known as FGF2) [22]. As the blood provides oxygen and nutrients to cells, angiogenesis has a critical role in cancer cells, which require high levels of oxygen and nutrients [23]. Therefore, angiogenesis inhibitors have been developed to block not only pathological angiogenesis but also tumor growth and metastasis.

Treatment with high concentrations of soluble PTK7 (sPTK7, the extracellular domain of PTK7) as a decoy receptor or a knockdown of *PTK7* inhibits VEGF-induced migration, invasion, tube formation, and angiogenesis in vivo [21]. PTK7 promotes KDR (also known as VEGFR2) oligomerization on the cell surface, resulting in enhanced sensitivity of KDR to VEGF [24]. Additionally, PTK7 can bind and activate FGFR1 independent of FGF [20]. In perivascular mononuclear cells, PTK7 induces the expression of KDR and ANGPT1 and consequently promotes angiogenesis by contributing to both neovascularization and vascular stabilization [25]. Therefore, PTK7 function inhibitors are expected to regulate angiogenesis.

To develop an agent that inhibits PTK7 function with in vivo stability, anti-PTK7 monoclonal antibodies (mAbs) were produced in this study. The ability of anti-PTK7 mAbs to neutralize PTK7-dependent angiogenic phenotypes was examined. The effects of anti-PTK7 mAbs on VEGF-induced adhesion, migration, invasion, and tube formation in human umbilical vein endothelial cells (HUVECs) in vitro and angiogenesis ex vivo and in vivo were analyzed. Additionally, the effects of anti-PTK7 mAbs on the VEGF-induced signaling pathway and the PTK7–KDR interaction were examined. The findings of this study indicate that anti-PTK7 mAbs inhibit angiogenesis and that they have potential therapeutic applications for impaired angiogenesis-associated diseases.

## 2. Materials and Methods

### 2.1. Cell Culture

Human embryonic kidney 293 (HEK293) cells were obtained from Korean Cell Line Bank (Seoul, Korea) and cultured in Dulbecco’s modified Eagle’s medium (Hyclone, South Logan, UT, USA) supplemented with 10% bovine serum (Gibco, Grand Island, NY, USA), 100 U/mL penicillin, and 100 μg/mL streptomycin. HUVECs were purchased from Zenbio (Durham, NC, USA) and cultured in Medium 199 (Gibco) supplemented with 20% fetal bovine serum (FBS; Hyclone), 5 U/mL heparin (Sigma-Aldrich, St. Louis, MO, USA), and 3 ng/mL human bFGF (Prospec, Ness-Ziona, Israel). HUVECs were used at passages 5–10 for the experiments. All cells were cultured at 37 °C, 5% CO_2_, and 95% air.

### 2.2. Antibodies

Antibodies were purchased from the following vendors: Santa Cruz Biotechnology (Santa Cruz, CA, USA), anti-phospho-ERK (sc-7383) and anti-FAK (sc-557) antibodies; Cell Signaling Technology (Beverly, MA, USA), anti-phospho-KDR (Tyr1175; 2478S), anti-KDR (2479S), anti-phospho-Src family (Tyr416; 2101S), anti-Src (2109S), anti-phospho-JNK (Thr183/Tyr185; 4668S), and anti-JNK (9252S) antibodies; Merck Millipore (Burlington, MA, USA), anti-phospho-FAK antibodies (Tyr396; abt135); Bioss (Boston, MA, USA), anti-ERK2 antibodies (bms-52068R); Sigma-Aldrich, anti-FLAG-M2 antibodies (F1804); BioLegend (San Diego, CA, USA), anti-HA antibodies (902302); Qiagen (Cambridge, MA, USA), anti-penta-His antibodies; AbClone (Seoul, Korea), anti-GAPDH antibodies (abc2003); KOMA Biotech (Seoul, Korea), horseradish peroxidase-conjugated goat anti-mouse-IgG and anti-rabbit-IgG antibodies. Rabbit anti-PTK7 anti-serum has been previously described [21].

### 2.3. Constructs Expressing sPTK7 and sPTK7 Domains

The pcDNA3-hPTK7-Ext-His construct encoding human sPTK7 (corresponding to human PTK7 Ig1-7-His) has been previously described [21]. The pcDNA3.1-hPTK7-Ig1-5-His, pcDNA3.1-hPTK7-Ig1-4.2-His, and pcDNA3.1-hPTK7-Ig1-3-His constructs encoding human PTK7-Ig1-5-His, PTK7-Ig1-4.2-His, and PTK7-Ig1-3-His, respectively, were generated by subcloning the complementary DNA (cDNA) fragments into pcDNA3.1. The cDNA fragments were generated using polymerase chain reaction (PCR) with the following primer pairs: Ig1-F and Ig5-His-R; Ig1-F and Ig4.2-His-R; and Ig1-F and Ig3-His-R (Table S1). Next, the cDNA fragments were digested with *EcoR*I and *Xba*I and ligated into the pcDNA3.1 vector that was digested with *EcoR*I and *Xba*I. The pcDNA3.1-hPTK7-Ig1-4-His construct encoding human PTK7-Ig1-4-His was generated using *Dpn*I-mediated deletion mutagenesis with the primer pair Ig1-4-His-F and Ig1-4-His-R (Table S2) and the pcDNA3.1-hPTK7-Ig1-4.2-His construct as a template. The pcDNA3.1-hPTK7-2-4-His and pcDNA3.1-hPTK7-Ig3-4-His constructs encoding human PTK7-Ig2-4-His and human PTK7-Ig3-4-His, respectively, were generated using *Dpn*I-mediated deletion mutagenesis with the primer pairs Ig2-4-His-F/Ig2-4-His-R and Ig3-4-His-F/Ig3-4-His-R, respectively (Table S2), and the hPTK7-Ig1-4-His construct as a template. All constructs were sequenced to confirm the absence of PCR errors. 

### 2.4. Expression and Purification of sPTK7 and sPTK7 Domains in HEK293 Cells

The expression constructs for His-tagged sPTK7 and sPTK7 domains were transfected into HEK293 cells using the calcium phosphate method, following a previously reported protocol [26]. To select the transfected cells, the cells were cultured in the presence of 1.2 mg/mL G418 (AG Scientific, San Diego, CA, USA) for 2 weeks. Stable single cell clones or mixed cell populations expressing His-tagged proteins were maintained in a medium with 0.6 mg/mL G418. Serum-free conditioned medium from cells stably expressing His-tagged proteins (for 4–5 days) was subjected to ammonium sulfate precipitation at 70% saturation. The pellets were dissolved in phosphate-buffered saline (PBS; 137 mM NaCl, 10 mM Na_2_HPO_4_, 2.7 mM KCl, and 2 mM KH_2_PO_4_; pH 7.4) containing 1 mM phenylmethylsulfonyl fluoride (PMSF) and 1 mM ethylenediaminetetraacetic acid (EDTA) and dialyzed against PBS. His-tagged proteins were purified using Ni^2+^-NTA agarose (Qiagen, Hilden, Germany).

### 2.5. Anti-PTK7 mAbs

Mouse anti-PTK7 hybridoma cell lines were established using purified human sPTK7 as the antigen (AbFrontier, Seoul, Korea). Anti-PTK7 mAbs were purified from ascites obtained via intraperitoneal injection of hybridomas into mice (AbClone).

### 2.6. Analysis of Binding Domains of Anti-PTK7 mAbs

Purified sPTK7-His and its deletion domains were incubated with anti-PTK7 mAbs at a 1:1 molar ratio for 2 h at 4 °C and pulled down with Ni^2+^-NTA agarose resins (Qiagen, Cambridge, MA, USA). Protein-bound resins were washed twice with PBS containing 0.1% Tween 20. Pulled-down proteins were resuspended in sodium dodecyl sulfate (SDS) sample buffer and subjected to Western blotting.

### 2.7. Western Blotting

Western blotting was performed as described previously [27]. Briefly, cell lysates or pull-down proteins were resuspended in SDS sample buffer and subjected to SDS-polyacrylamide gel electrophoresis (SDS-PAGE). The resolved proteins were transferred to a polyvinylidene difluoride membrane (Millipore, Bedford, MA, USA). The membrane was incubated with the indicated antibodies. Immunoreactive signals were developed using Immobilon Western Chemiluminescent HRP Substrate (Millipore, Bedford, MA, USA) and AMERSHAM ImageQuant 800 (Cytiva, Marlborough, MA, USA).

### 2.8. Adhesion Assay

HUVECs were starved for 6 h in M199 medium containing 1% FBS and the cells were resuspended in the same medium. The cell suspension (1 × 10^4^ cells/100 μL) was pre-treated with anti-PTK7 mAbs (10 μg/mL) or human sPTK7 (4 μg/mL) for 30 min at 25 °C and loaded into gelatin-coated 96-well plates. Then, the cells were incubated with 10 ng/mL human VEGF (KOMA Biotech) for 1 h. Next, the cells were fixed with 3.7% paraformaldehyde in PBS and stained with 0.005% crystal violet. The stained cells were lysed with 1% SDS. The absorbance of the mixture at 600 nm was measured.

### 2.9. Wound Healing Assay

The monolayer of HUVECs grown in 12-well plates was starved in the M199 medium containing 1% FBS for 6 h. A scratch was introduced into the monolayer using a micropipette tip. The cells were washed to remove debris and pre-treated with anti-PTK7 mAbs (10 μg/mL) or human sPTK7 (4 μg/mL) in M199 medium with 1% FBS. Next, the cells were incubated with 10 ng/mL human VEGF for 14 h and observed under a light microscope.

### 2.10. Chemotactic Migration and Invasion Assay

HUVECs were starved in the M199 medium containing 1% FBS for 6 h. Chemotactic migration and invasion assays were performed as described previously [21] with minor modifications. HUVECs were pre-treated with anti-PTK7 mAbs (3 and 10 μg/mL) or human sPTK7 (4 μg/mL) for 30 min at 25 °C prior to loading the cells into the upper compartment of Transwell. Cells that migrated to the bottom surface of the filter were fixed with 3.7% paraformaldehyde in PBS, stained with 0.02% crystal violet, and analyzed under a light microscope (Olympus, Tokyo, Japan). The stained cells were lysed with 1% SDS and the absorbance at 600 nm of the mixture was measured.

### 2.11. Capillary-like Tube Formation Assay

Capillary-like tube formation assays were performed as previously described [28]. Briefly, HUVECs were starved in M199 medium containing 1% FBS for 6 h, harvested with trypsin, and resuspended in the same medium. Cells (2 × 10^5^ cells/well of a 24-well plate) were pre-treated with anti-PTK7 mAbs (10 μg/mL) or sPTK7 (4 μg/mL) for 30 min and loaded on a 24-well plate whose wells were pre-coated with 300 μL of growth-factor-reduced Matrigel (Corning, Bedford, MA, USA). The cells were then incubated with a final 20 ng/mL human VEGF at 37 °C for 16 h and analyzed using light microscopy. The number and length of capillary-like tubes were quantified using ImageJ angiogenesis analyzer [29].

### 2.12. Mouse Aortic Ring Assay

The aortic ring assay was performed as described previously [30]. The thoracic aorta of mice (aged 6–7 weeks) was transferred to a Petri dish filled with cold PBS, and the surrounding fat tissue was removed. The aorta was sliced using a surgical blade and placed in the center of solidified growth-factor-reduced Matrigel (150 μL). The samples were incubated for 20 min at 37 °C in a 48-well dish. Additional Matrigel (150 μL) was added to the top of each ring, and the samples were incubated at 37 °C for 20 min. M199 medium with 1% FBS (200 μL) containing 20 ng/mL mouse VEGF (KOMA Biotech) with or without anti-PTK7 mAbs (10 μg/mL) or sPTK7 (4 μg/mL) was added to each well. This medium was replaced once every 5 days. After 12 days, the outgrowth of cells from the aorta was analyzed under a phase-contrast microscope. The sprouting area extending from a constant length of the aorta was quantified using ImageJ.

### 2.13. Matrigel Plug Assay

The Matrigel plug assay was performed as described previously [24]. Briefly, growth-factor-reduced Matrigel (0.5 mL) containing 32 U heparin and 250 ng of mouse VEGF or mouse VEGF plus anti-PTK7 mAbs (3 and 10 μg/mL) was subcutaneously injected into female C57BL/6 mice aged 4 weeks. After 12 days, the mice were sacrificed and the plugs were recovered. The hemoglobin (Hb) content in the plugs was measured using Drabkin’s reagent kit 525 (Sigma-Aldrich) to quantify blood vessel formation.

### 2.14. Analysis of Signaling Proteins in HUVECs

Subconfluent HUVECs were starved in the M199 medium supplemented with 1% FBS for 6 h. The cells were pre-incubated with anti-PTK7 mAbs (10 μg/mL) or sPTK7 (4 μg/mL) for 30 min. Next, the cells were stimulated with 10 ng/mL human VEGF for 2 min to analyze receptor phosphorylation, 1 h to analyze the phosphorylation of FAK [31,32], or 10 min to analyze the phosphorylation of other signaling molecules. The cells were then lysed with radioimmunoprecipitation assay lysis buffer [33] containing 1 mM Na_3_VO_4_ and 5 mM NaF. Cell lysates were resuspended in SDS sample buffer and subjected to Western blotting. Protein bands from Western blots were quantified with ImageJ.

### 2.15. Co-Expression of PTK7 and KDR in HEK293 Cells

The lentiviral transfer vector pHRST-hPTK7-FLAG-IRES-eGFP encoding human PTK7 with a C-terminal FLAG tag has been previously described [34]. Lentiviruses expressing PTK7-FLAG were propagated in HEK293T cells by co-transfection of pHRST-hPTK7-FLAG-IRES-eGFP, a packaging vector psPAX2, and an envelope vector pMD2.G (Addgene, Cambridge, MA, USA) as previously described [17]. Subconfluent HEK293 cells were infected with PTK7-FLAG lentiviruses. HEK293 cells expressing PTK7-FLAG were transfected with pcDNA3.1-Kozak-KDR encoding human KDR with a C-terminal HA tag [24] using the calcium phosphate method.

### 2.16. Pull-Down Assay

Subconfluent HEK293 cells co-expressing PTK7-FLAG and KDR-HA were incubated with anti-PTK7 mAbs (10 μg/mL) or sPTK7 (4 μg/mL) for 2 h. Cells were lysed with NP-40 lysis buffer (50 mM Tris–HCl (pH 7.4), 150 mM NaCl, and 1% NP-40) containing 5 mM NaF, 1 mM Na_3_VO_4_, and protease inhibitor cocktail III (Calbiochem, La Jolla, CA, USA). The lysates were incubated with mouse anti-FLAG M2 antibodies (Sigma-Aldrich) for 2 h. The protein-bound resins were then washed with NP-40 lysis buffer. Pulled-down proteins were resuspended in SDS sample buffer and subjected to Western blotting.

### 2.17. Statistical Analysis

The means between the two groups were compared using Student’s *t*-test. All data obtained from at least three independent experiments are expressed as mean ± standard deviation. Differences were considered significant at *p* < 0.05.

## 3. Results

### 3.1. Analysis of the PTK7-Binding Domains of Anti-PTK7 mAbs

**Figure 1:**
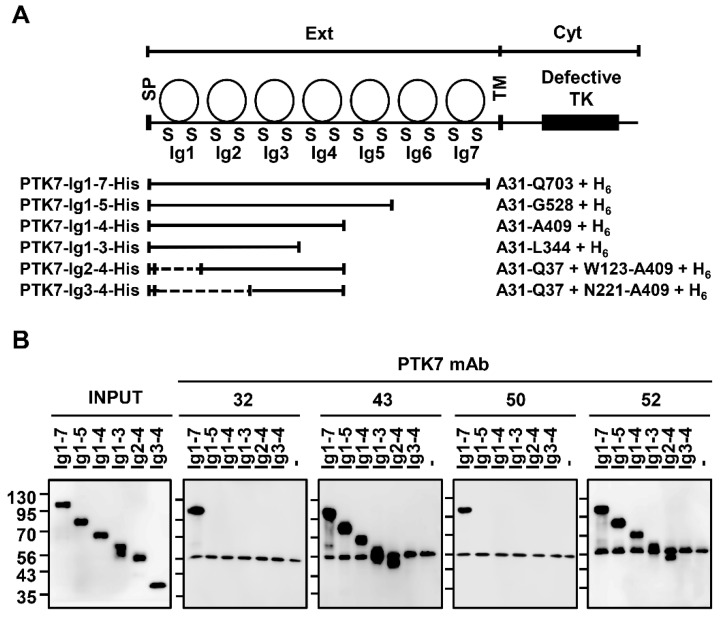
Analysis of PTK7-binding domains of anti-PTK7 mAbs. (**A**) Diagrams of PTK7 and its deletion mutants (PTK7-Ig1-7-His also called sPTK7, PTK7-Ig1-5-His, PTK7-Ig1-4-His, PTK7-Ig1-3-His, PTK7-Ig2-4-His, and PTK7-Ig3-4-His) are shown. SP, signal peptide; Ext, extracellular region including 7 Ig domains; TM, transmembrane domain; Cyt, cytosolic region containing a catalytically defective catalytic tyrosine kinase domain (indicated as Defective TK); and H_6_, His tag. (**B**) Results of the pull-down assay to determine the PTK7-binding domains of mAb-32, mAb-43, mAb-50, and mAb-52. PTK7-Ig1-7-His and its deletion mutants were incubated with anti-PTK7 mAbs at a 1:1 molar ratio. Proteins pulled down with Ni^2+^-NTA agarose resins were subjected to SDS-PAGE and Western blotting with anti-His antibodies.

To analyze the PTK7-binding domains of anti-PTK7 mAbs, a pull-down assay was performed using anti-PTK7 mAbs. The following deletion mutants of the extracellular region of PTK7 (sPTK7; named as PTK7-Ig1-7-His for comparison) were used for the analysis: PTK7-Ig1-5-His, PTK7-Ig1-4-His, PTK7-Ig1-3-His, PTK7-Ig2-4-His, and PTK7-Ig3-4-His (Figure 1A). mAb-32 and mAb-50, which bind to PTK7-Ig1-7-His but not to other deletion mutants, recognize PTK7 Ig6–7 domains. Meanwhile, mAb-43 and mAb-52, which bind to PTK7-Ig1-7-His, PTK7-Ig1-5-His, PTK7-Ig1-4-His, PTK7-Ig1-3-His, and PTK7-Ig2-4-His but not to PTK7-Ig3-4-His, recognize the PTK7 Ig2 domain (Figure 1B and Figure S2).

### 3.2. Effect of Anti-PTK7 mAbs on Angiogenic Phenotypes in HUVECs

**Figure 2:**
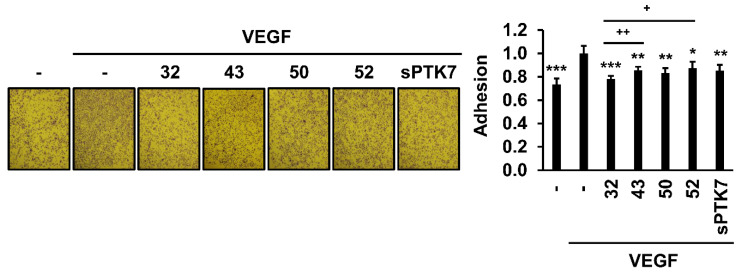
Effect of anti-PTK7 mAbs on the adhesion of HUVECs. Adhesion of HUVECs was assessed for 1 h in the presence of anti-PTK7 mAbs (mAb-32, mAb-43, mAb-50, and mAb-52; 10 μg/mL) or sPTK7 (4 μg/mL) after plating the cells on a 96-well plate whose wells were pre-coated with 0.1% gelatin. The number of adhered cells was measured after crystal violet staining. Representative images are shown. Magnification = ×40. “–”: no treatment with anti-PTK7 mAb or sPTK7. The graph shows the number of adhered cells in the mAb-treated groups relative to that in the VEGF-alone group (n = 5). Data represent mean ± standard deviation. * *p <* 0.05, ** *p <* 0.01, and *** *p <* 0.001 vs. VEGF-alone control. + *p <* 0.05 and ++ *p <* 0.01 vs. mAb-32-treated group. The absence of the *p* value between antibody-treated groups indicates statistical insignificance.

**Figure 3:**
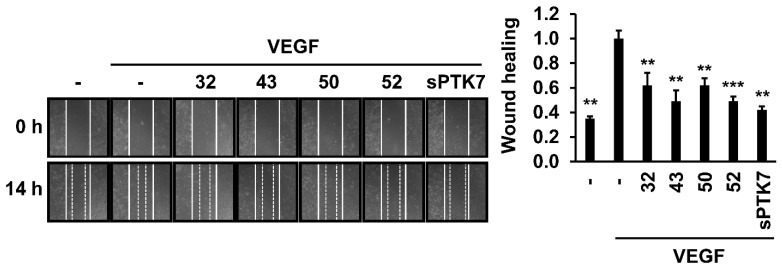
Effect of anti-PTK7 mAbs on wound healing in the HUVEC monolayer. The monolayer of HUVECs was scratched, and the cells were incubated with anti-PTK7 mAbs (mAb-32, mAb-43, mAb-50, and mAb-52; 10 μg/mL) or sPTK7 (4 μg/mL). Micrographs were captured at 0 and 14 h. Representative images are shown. Magnification = ×40. “–”: no treatment with anti-PTK7 mAb or sPTK7. The graph shows the migration of cells to the wound area in the mAb-treated and sPTK7-treated groups relative to that in the VEGF-alone group (n = 3). Data are represented as mean ± standard deviation. ** *p <* 0.01 and *** *p <* 0.001 vs. VEGF-alone control. The absence of the *p* value between antibody-treated groups indicates statistical insignificance.

**Figure 4:**
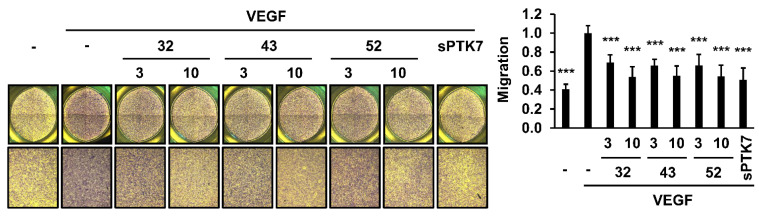
Effect of anti-PTK7 mAbs on the chemotactic migration of HUVECs. Chemotactic migration of cells treated with mAb-32, mAb-43, and mAb-52 (3 and 10 μg/mL) or sPTK7 (4 μg/mL) was analyzed for 4 h. Cells that migrated to the bottom surface of the well were counted after crystal violet staining. Magnification = ×40. “–”: no treatment with anti-PTK7 mAb or sPTK7. The graph shows the number of migrated cells in the mAb-treated and sPTK7-treated groups relative to that in the VEGF-alone group (n = 6). Data are represented as mean ± standard deviation. *** *p <* 0.001 vs. VEGF-alone control. The absence of the *p* value between antibody-treated groups indicates statistical insignificance.

**Figure 5:**
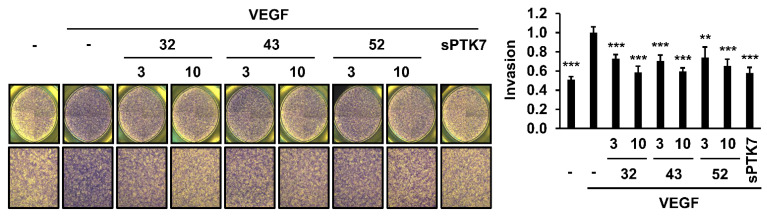
Effect of anti-PTK7 mAbs on the chemotactic invasion of HUVECs. Chemotactic invasion of cells treated with mAb-32, mAb-43, and mAb-52 (3 and 10 μg/mL) or sPTK7 (4 μg/mL) was analyzed for 24 h using Transwell chambers. Cells that invaded the bottom surface of the well were counted after crystal violet staining. Magnification = ×40. “–”: no treatment with anti-PTK7 mAb or sPTK7. The graph shows the invasion of cells in the mAb-treated and sPTK7-treated groups relative to that in the VEGF-alone group (n = 6). Data are represented as mean ± standard deviation. ** *p <* 0.01 and *** *p <* 0.001 vs. VEGF-alone control. The absence of the *p* value between antibody-treated groups indicates statistical insignificance.

The effect of anti-PTK7 mAbs on angiogenic phenotypes (including adhesion, wound healing, chemotactic migration, and invasion) in HUVECs was analyzed. As high concentrations of sPTK7 inhibit angiogenic phenotypes [21,24], sPTK7 (4 μg/mL) was used as a positive control to inhibit PTK7 functions. mAb-32, mAb-43, mAb-50, mAb-52 (10 μg/mL each), and sPTK7 decreased the VEGF-induced adhesion of HUVECs to 78.2% ± 2.5%, 85.5% ± 3.1%, 83.2% ± 4.0%, 87.3% ± 5.5%, and 85.3 ± 5.0%, respectively (Figure 2). mAb-32, mAb-43, mAb-50, mAb-52 (10 μg/mL each), and sPTK7 also decreased the VEGF-induced wound healing in the HUVEC monolayer to 62.1% ± 9.9%, 49.0% ± 8.8%, 62.1% ± 5.4%, 49.1% ± 3.8%, and 42.0% ± 3.0%, respectively (Figure 3). Additionally, mAb-32, mAb-43, and mAb-52 dose-dependently suppressed the VEGF-induced chemotactic migration in HUVECs. At a concentration of 10 μg/mL, mAb-32, mAb-43, mAb-52, and sPTK7 decreased the VEGF-induced chemotactic migration of HUVECs to 53.8% ± 10.1%, 55.1% ± 10.2%, 54.5% ± 11.9%, and 50.8% ± 12.4%, respectively (Figure 4). Furthermore, mAb-32, mAb-43, and mAb-52 dose-dependently suppressed the VEGF-induced invasion of HUVECs. At a concentration of 10 μg/mL, mAb-32, mAb-43, mAb-52, and sPTK7 decreased the VEGF-induced invasion of HUVECs to 58.6% ± 6.2%, 59.7% ± 3.5%, 65.2% ± 7.2%, and 57.8% ± 5.9%, respectively (Figure 5). The ability of different anti-PTK7 mAbs to inhibit migration, wound healing, and invasion was not significantly different. However, the adhesion of the mAb-32-treated group was significantly lower than that of the mAb-43-treated and mAb-52-treated groups (Figure 2). Furthermore, mAb-32, mAb-43, mAb-50, mAb-52 (10 μg/mL), and sPTK7 (4 μg/mL) did not exert cytotoxic effects on HUVECs (Figure S1). Therefore, the anti-PTK7 mAbs that we tested significantly inhibited VEGF-induced angiogenic phenotypes in HUVECs.

### 3.3. Effect of Anti-PTK7 mAbs on Angiogenesis In Vitro and Ex Vivo

**Figure 6:**
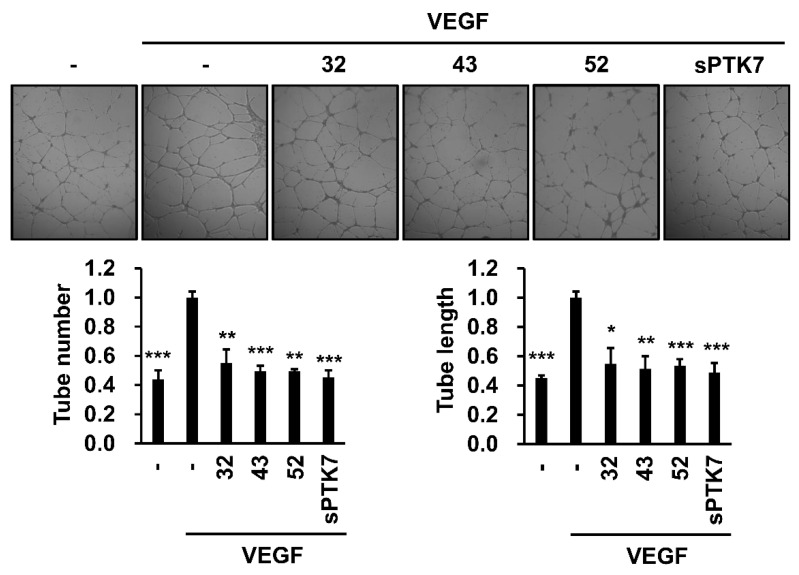
Effect of PTK7 mAbs on VEGF-induced angiogenesis in vitro. Capillary-like tube formation assay was performed with HUVECs to evaluate in vitro angiogenesis. HUVECs were starved in M199 medium with 1% FBS for 6 h. The cells were plated on solidified growth-factor-reduced Matrigel and incubated with or without VEGF (20 ng/mL) in the presence of mAb-32, mAb-43, and mAb-52 (10 μg/mL) or sPTK7 (4 μg/mL) at 37 °C for 16 h. Representative images are shown (magnification = ×40). “–”: no treatment with anti-PTK7 mAb or sPTK7. The number and length of tubes was quantified using the ImageJ angiogenesis analyzer. The graphs show the tube number and the tube length in the mAb-treated and sPTK7-treated groups relative to that in the VEGF-alone group (n = 3). Data are represented as mean ± standard deviation. * *p <* 0.05, ** *p <* 0.01 and *** *p <* 0.001 vs. VEGF-alone control. The absence of the *p* value between antibody-treated groups indicates statistical insignificance.

The effect of anti-PTK7 mAbs on angiogenesis in vitro was analyzed using the capillary-like tube formation assay. VEGF (20 ng/mL) induced capillary-like tube formation in HUVECs cultured on Matrigel. mAb-32, mAb-43, mAb-52 (10 μg/mL), and sPTK7 (4 μg/mL) significantly decreased the VEGF-induced tube formation to 55.2% ± 9.3%, 49.4% ± 3.8%, 49.4% ± 1.3%, and 45.2% ± 5.0% based on tube numbers, and to 54.7% ± 10.8%, 51.4% ± 8.5%, 66.9% ± 11.4%, and 48.9% ± 6.5% based on tube length, respectively (Figure 6).

**Figure 7:**
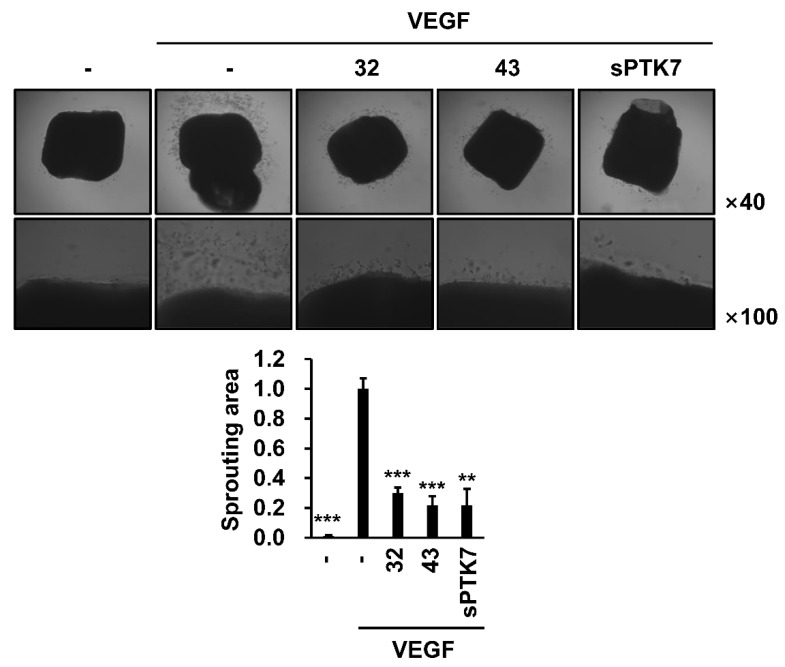
Effect of PTK7 mAbs on VEGF-induced angiogenesis ex vivo. Mouse aortic ring assay was performed to evaluate angiogenesis ex vivo. Mouse aortas were placed on solidified growth-factor-reduced Matrigel and incubated in M199 medium with 1% FBS and with or without mouse VEGF (20 ng/mL) in the presence of mAb-32 or mAb-43 (10 μg/mL) or sPTK7 (4 μg/mL) for 12 days. Outgrowth of endothelial cells from aortas was observed under a phase-contrast microscope (magnification = ×40 and ×100). “–”: no treatment with anti-PTK7 mAb or sPTK7. The graph shows the sprouting area from the aorta in the mAb-treated and sPTK7-treated groups relative to that in the VEGF-alone group (n = 3). Data are represented as mean ± standard deviation. ** *p <* 0.01 and *** *p <* 0.001 vs. VEGF-alone control. The absence of the *p* value between antibody-treated groups indicates statistical insignificance.

The effect of anti-PTK7 mAbs on ex vivo angiogenesis was evaluated using the mouse aortic ring assay. Treatment with 20 ng/mL VEGF induced sprouting and outgrowth of endothelial cells from the aortas. The endothelial cell outgrowth in the mAb-32-, mAb-43- (10 μg/mL), and sPTK7 (4 μg/mL)-treated groups was inhibited to 29.9% ± 3.7%, 21.6% ± 6.3%, and 21.7% ± 11.1%, respectively (Figure 7).

### 3.4. Effect of Anti-PTK7 mAbs on Angiogenesis In Vivo

**Figure 8:**
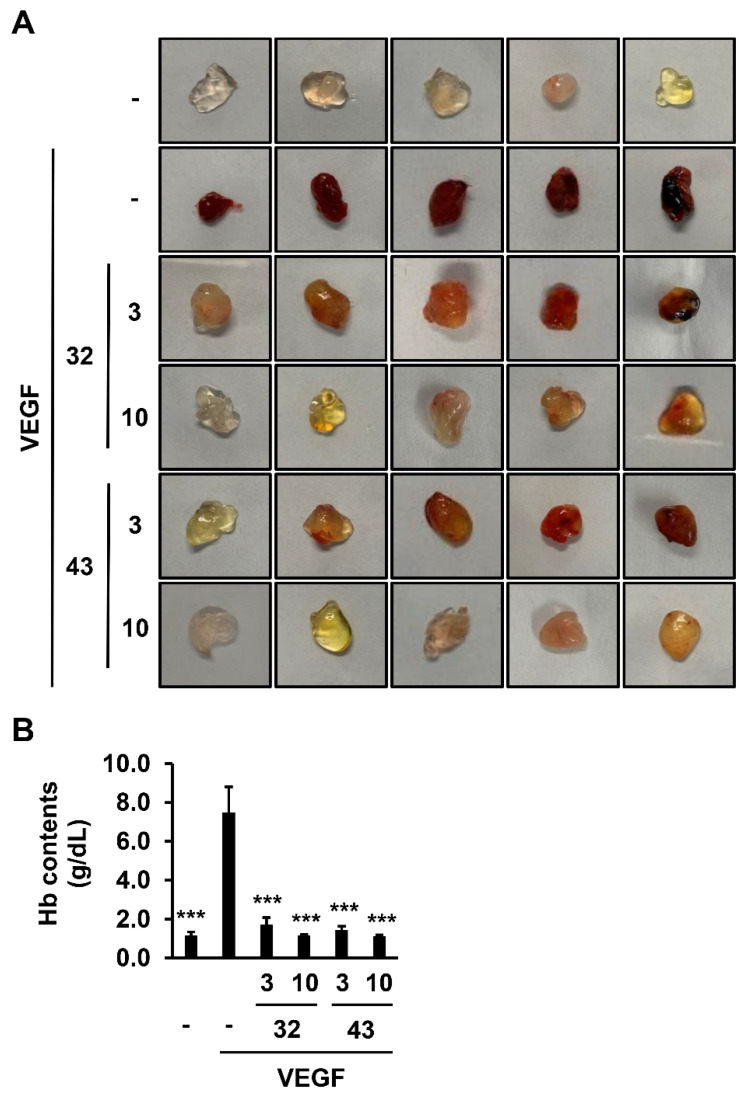
Effect of PTK7 mAbs on VEGF-induced angiogenesis in vivo. Matrigel plug assay was performed to analyze angiogenesis in vivo. Mice were subcutaneously injected with growth-factor-reduced Matrigel (0.5 mL) with or without mouse VEGF (500 ng/mL) in the presence of mAb-32 or mAb-43 (3 or 10 μg/mL). After 12 days, the mice were sacrificed and the plugs were recovered. (**A**) Images of the plugs are shown (n = 5). “–”: no treatment with anti-PTK7 mAb. (**B**) Hemoglobin content in the plugs was measured using Drabkin’s Reagent Kit 525 to quantify the degree of angiogenesis. Data are represented as mean ± standard deviation. *** *p <* 0.001 vs. VEGF-alone control. The absence of the *p* value between antibody-treated groups indicates statistical insignificance.

To examine the effect of anti-PTK7 mAbs on angiogenesis in vivo, a Matrigel plug assay was performed. Treatment with mouse VEGF resulted in plugs with a dark red color, indicating induction of angiogenesis. Co-treatment with 3 μg/mL mAb-32 or mAb-43 and VEGF resulted in plugs with an orange or pale red color. In contrast, co-treatment with 10 μg/mL of mAb-32 or mAb-43 and VEGF resulted in plugs with a white or yellow color (Figure 8A). The degree of in vivo angiogenesis was quantified by measuring the hemoglobin (Hb) content in the plugs. The Hb content in the plugs retrieved from mice treated with VEGF was 7.48 ± 1.33 g/dL. However, co-treatment with 3 or 10 μg/mL mAb-32 and VEGF decreased the Hb levels to 1.73 ± 0.36 or 1.15 ± 0.49 g/dL, respectively. Co-treatment with 3 or 10 μg/mL mAb-43 and VEGF also decreased the Hb levels to 1.44 ± 0.19 or 1.13 ± 0.06 g/dL, respectively (Figure 8B). Thus, mAb-32 and mAb-43 concentration-dependently inhibited VEGF-induced angiogenesis in vivo.

### 3.5. Effect of Anti-PTK7 mAbs on VEGF-Induced KDR Signaling in HUVECs

**Figure 9:**
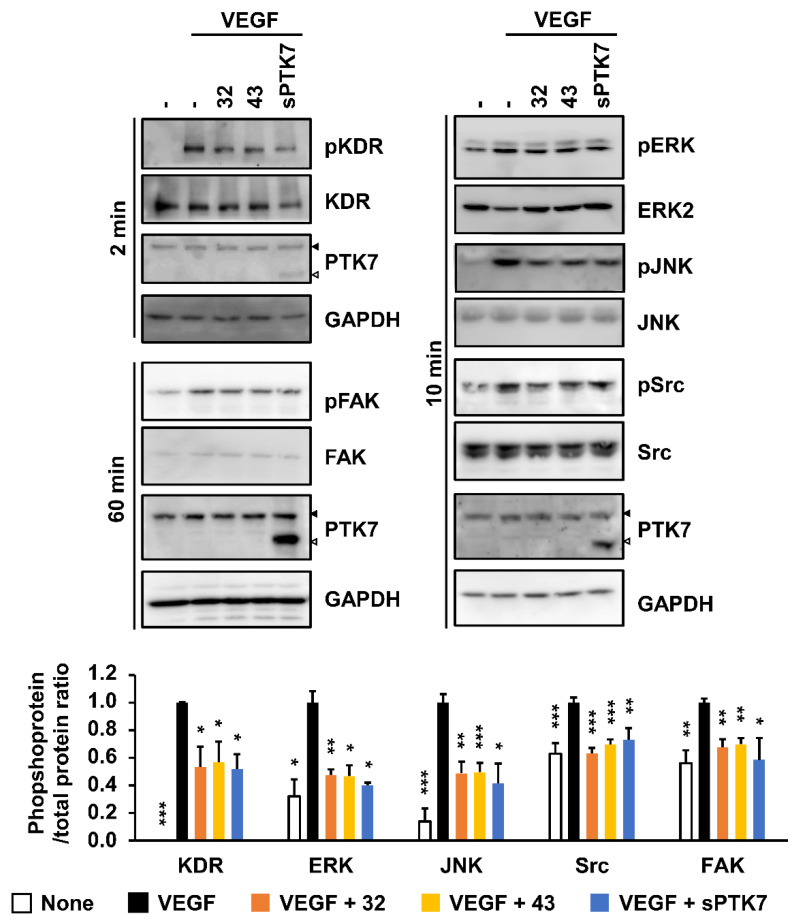
Effect of PTK7 mAbs on VEGF-induced activation of KDR and its downstream signaling proteins in HUVECs. HUVECs were starved in M199 medium with 1% FBS for 6 h and pre-treated with mAb-32 or mAb-43 (10 μg/mL) or sPTK7 (4 μg/mL) 30 min before VEGF stimulation. The cells were stimulated with 10 ng/mL VEGF for 2 min to analyze the phosphorylation of KDR, 60 min to analyze the phosphorylation of FAK, or 10 min to analyze the phosphorylation of other signaling proteins. The phosphorylation of KDR and its downstream signaling proteins was examined using Western blotting. Solid and open triangles indicate full-length PTK7 and sPTK7, respectively. “–”: no treatment with anti-PTK7 mAb or sPTK7. The graph shows the phosphoprotein/total protein ratio in the mAb-treated and sPTK7-treated groups relative to that in the VEGF-alone group (n = 3). Data are represented as mean ± standard deviation. * *p <* 0.05, ** *p <* 0.01 and *** *p <* 0.001 vs. VEGF-alone control. The absence of the *p* value between antibody-treated groups indicates statistical insignificance.

Angiogenesis is mediated by various signaling pathways, including ERK and JNK signaling pathways involved in cell proliferation and differentiation and FAK and Src signaling pathways involved in cell adhesion and migration [35,36]. Therefore, the effect of anti-PTK7 mAbs on the VEGF-induced activation of signaling proteins in HUVECs was examined. mAb-32, mAb-43 (10 μg/mL each), and sPTK7 (4 μg/mL) downregulated the phosphorylation of KDR to 53.2% ± 14.8%, 56.9% ± 14.9%, and 51.8% ± 10.7%, respectively. In addition, mAb-32, mAb-43, and sPTK7 reduced the phosphorylation of ERK to 47.6% ± 4.0%, 7.8% ± 14.9%, and 40.2% ± 1.7%; the phosphorylation of JNK to 48.7% ± 8.5%, 50.0% ± 6.6%, and 41.4% ± 14.5%; the phosphorylation of Src to 63.3% ± 3.8%, 69.8% ± 3.7%, and 73.0% ± 8.4%; and the phosphorylation of FAK to 67.8% ± 5.7%, 69.8% ± 4.4%, and 58.5% ± 15.9%, respectively (Figure 9 and Figure S3). These results indicate that anti-PTK7 mAbs downregulate the activation of VEGF-induced KDR and downstream signaling pathways involved in angiogenesis.

### 3.6. Effect of Anti-PTK7 mAbs on PTK7–KDR Interaction

**Figure 10:**
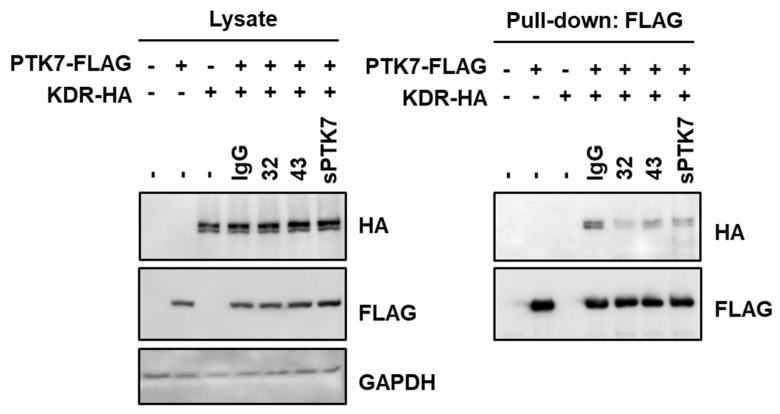
Effect of PTK7 mAbs on PTK7-KDR interaction. HEK293 cells overexpressing PTK7-FLAG and KDR-HA were incubated with mAb-32 or mAb-43 (10 μg/mL) for 2 h. Bovine IgG (10 μg/mL) and sPTK7 (4 μg/mL) were used as negative and positive controls, respectively. Cell lysates and proteins pulled down with anti-FLAG antibodies were subjected to SDS-PAGE and Western blotting with anti-HA antibodies to detect KDR, anti-FLAG antibodies to detect PTK7, and anti-GAPDH antibodies. “+” and “–” for PTK7-FLAG or KDR-HA indicate expression and no expression of PTK7-FLAG or KDR-HA, respectively. “–” just above the western blot indicates no treatment with anti-PTK7 mAb or sPTK7.

Next, the effect of anti-PTK7 mAbs on PTK7–KDR interaction was examined. PTK7–KDR interaction in HEK293 cells expressing PTK7 and KDR was analyzed by co-precipitation of KDR-HA with PTK7-FLAG after treatment with mAb-32 or mAb-43. Previously, we had reported that sPTK7 (4 μg/mL) decreased the PTK7–KDR interaction by competing with PTK7 [21,24]. Compared with bovine IgG (10 μg/mL), sPTK7 inhibited the binding of PTK7 to KDR. Under these conditions, mAb-32 and mAb-43 (10 μg/mL) decreased the binding of PTK7 to KDR (Figure 10 and Figure S4). These results indicate that anti-PTK7 mAbs downregulate VEGF-induced KDR activation and its downstream signaling pathways by inhibiting PTK7–KDR interaction.

## 4. Discussion

As PTK7 is reported to be involved in oncogenesis and angiogenesis, it is a potential therapeutic target for cancer and vascular diseases. However, a small-molecule tyrosine kinase inhibitor targeting PTK7 cannot be designed as PTK7 is a catalytically defective RPTK. To suppresses the oncogenic function of ERBB3 (also known as HER3), which is another catalytically defective RPTK, an anti-ERBB3 antibody was developed and is currently undergoing clinical trials as an anti-cancer drug [37]. Previously, we had demonstrated that treatment with sPTK7 as a decoy receptor or *PTK7* knockdown inhibits the angiogenic functions of endothelial cells and angiogenesis in vivo [21]. Additionally, PTK7 enhanced VEGF-induced angiogenesis by promoting KDR oligomerization [24]. As the inhibition of PTK7 function was reported to suppress angiogenesis, this study developed anti-sPTK7 mAbs and evaluated their effects on the angiogenesis-related functions of PTK7.

To determine the PTK7 subdomains to which the selected anti-PTK7 mAbs (mAb-32, mAb-43, mAb-50, and mAb-52) bind, this study constructed deletion mutants of PTK7 and evaluated their abilities to bind to mAbs. mAb-43 and mAb-52 recognized the PTK7 Ig2 domain, whereas mAb-32 and mAb-50 recognized the PTK7 Ig6–7 domains.

Angiogenesis involves adhesion, migration, and invasion following chemotaxis of endothelial tip cells [38]. Previously, we had reported that PTK7 can enhance the angiogenic phenotypes of endothelial cells, such as wound healing, chemotactic migration, and invasion [24]. mAb-32, mAb-43, mAb-50, and mAb-52 inhibited VEGF-induced adhesion and wound healing in HUVECs. Additionally, mAb-32, mAb-43, and mAb-52 dose-dependently decreased VEGF-induced chemotactic migration and invasion in HUVECs.

It was known that endothelial cells can differentiate into a tubular structure, mimicking a capillary, on Matrigel in vitro [39]. However, since lumens are rarely found in this structure, it was proposed to express this structure in terms of cords rather than tubes [40]. Nevertheless, we used this assay because it reproducibly demonstrates the migration and differentiation of endothelial cells and referred to it herein as capillary-like tube formation because it is widely used by that name. mAb-32, mAb-43, and mAb-52 decreased VEGF-induced capillary-like tube formation in HUVECs. The aortic ring assay enables ex vivo analysis of angiogenic processes, including migration, tube formation, and microvascular branching [41,42]. The Matrigel plug assay, an in vivo assay, enables the analysis of newly formed blood vessels in Matrigel plugs transplanted into mice [43]. The results of the mouse aortic ring assay revealed that mAb-32 and mAb-43 significantly inhibited VEGF-induced sprouting of endothelial cells. Furthermore, the results of the Matrigel plug assay revealed that mAb-32 and mAb-43 significantly inhibited VEGF-induced blood vessel formation in mice. Therefore, this study demonstrated that mAb-32 and mAb-43 inhibit angiogenesis ex vivo and in vivo.

Dana and colleagues reported that PTK7 knockdown decreased VEGF-induced phosphorylation of FLT-1 but not KDR, in mouse endothelial MS1 cells, and that PTK7 interacted with FLT-1 but not with KDR or FLT-4 [44]. In contrast, we found that treatment of sPTK7 (4 μg/mL) or knockdown of PTK7 in HUVECs inhibited VEGF-induced phosphorylation of KDR but not FLT-1. In HUVECs, as well as HEK293 cells expressing PTK7 and KDR or FLT-1, KDR was co-precipitated with PTK7 but FLT-1 was not. Therefore, it is evident that PTK7 plays an important role in angiogenesis by regulating the activation of KDR rather than FLT-1 and activating downstream signaling proteins such as ERK, JNK, FAK, and Src [24]. Consistently, these downstream signaling proteins are known to be involved in the proliferation, survival, adhesion, and migration of endothelial cells [45,46,47]. Treatment with anti-PTK7 mAbs downregulated the VEGF-induced phosphorylation of KDR, ERK, JNK, FAK, and Src, and the phosphorylation levels were comparable to those in the sPTK7 (4 μg/mL)-treated group.

To examine the mechanism underlying mAb-mediated inhibition of KDR activation, the binding of PTK7 to KDR was examined in the presence of anti-PTK7 mAbs. mAb-32 and mAb-43 inhibited the interaction between PTK7 and KDR. Previously, we had demonstrated that PTK7 promotes KDR activation by inducing KDR oligomerization [24]. Additionally, VEGF binds to the KDR Ig2–3 domains and promotes a homotypic interaction of the KDR Ig4–7 domains, resulting in the phosphorylation of the kinase [48]. As mAb-32 and mAb-43 bind to the PTK7 Ig2 and Ig6–7 domains, respectively, anti-PTK7 mAbs can impair PTK7–KDR interaction due to steric hindrance.

Tumorigenesis can be inhibited by blocking angiogenesis. Several anti-angiogenic drugs that specifically target the VEGF signaling pathway have been developed [49], and their therapeutic effects have been demonstrated in various types of cancer. For example, sunitinib (Sutent, Pfizer, New York, NY, USA), sorafenib (Nexavar, Bayer, Leverkusen, Germany), and pazopanib (Votrient, GSK, Brentford, UK) are small-molecular-weight inhibitors of receptor tyrosine kinases, including VEGFRs [50,51,52]. Bevacizumab (Avastin, Genentech, San Francisco, CA, USA) is a recombinant humanized mAb that targets VEGF-A and inhibits the formation of the VEGF/KDR complex [53].

Whereas endothelial cells have decreased somatic mutation burdens, tumor endothelial cells exhibit increased genetic instability owing to angiogenic factors, cytokines, and growth factors secreted into the tumor microenvironment [54,55]. Therefore, anti-angiogenic drugs have been successful to some extent. Nonetheless, anti-angiogenesis therapies are associated with several limitations, such as the unresponsiveness of tumor cells to drugs and induction of tumor invasiveness [56,57]. For example, clinical doses of sunitinib induce metastasis and invasiveness in several tumors [58]. The anti-tumor efficacy of bevacizumab was low in some cell types despite an adequate initial response to the treatment [59,60]. Therefore, we propose that anti-PTK7 neutralizing antibodies, which indirectly regulate KDR activation, are potential alternative therapeutics for anti-angiogenesis therapy.

In addition to its role in KDR activation, PTK7 enhances FGFR1 activation in the absence of ligands or the presence of aFGF or bFGF [20]. Therefore, mAbs that inhibit PTK7 function may interfere with the interaction of PTK7 with FGFR1. The FGF-mediated signaling pathway plays an important role in angiogenesis [61]. Thus, anti-PTK7 mAbs, which can block both VEGF-mediated and FGF-mediated signaling, may exert a potent inhibitory effect on angiogenesis. Additionally, PTK7 enhances signaling pathways involving ROR2 [5] and EGFR [62] and is involved in non-canonical and canonical Wnt signaling pathways [9]. As proteins that interact with PTK7 are still being discovered, applications of anti-PTK7 neutralizing mAbs can be further expanded.

In addition to anti-angiogenic drugs, including mAbs and tyrosine kinase inhibitors, antibody–drug conjugates (ADCs) that target tumor vasculature have been reported [63,64]. PF-06647020, a humanized anti-PTK7 ADC, has been developed and is currently being evaluated in clinical trials for the treatment of ovarian cancer, triple-negative breast cancer, and non-small cell lung cancer [65,66]. Additionally, PF-06647020 acts on endothelial cells and inhibits the sprouting of blood vessels [67]. However, the limitations of ADCs include off-target cytotoxicity [68]. Therefore, we suggest that anti-PTK7 neutralizing mAbs exert therapeutic effects on aberrant angiogenesis-related diseases, including metastatic cancer, by specifically inhibiting PTK7 functions.

## 5. Conclusions

PTK7, a catalytically defective RPTK, promotes angiogenesis by activating catalytically active RPTKs, such as KDR and FGFR1. To regulate angiogenesis by blocking PTK7 function, this study developed anti-PTK7 mAbs and analyzed their effects on angiogenesis. The selected anti-PTK7 mAbs inhibited VEGF-induced angiogenic phenotypes, including endothelial cell adhesion, migration, invasion, and tube formation, as well as angiogenesis ex vivo and in vivo. Additionally, anti-PTK7 mAbs downregulated the VEGF-induced activation of KDR and its downstream signaling proteins and inhibited the interaction of PTK7 with KDR. These results suggest that anti-PTK7 neutralizing mAbs have potential applications for the treatment of aberrant angiogenesis-associated diseases, such as cancer metastasis.

## Data Availability

Data are contained within the article and Supplementary Materials.

## Supplementary Materials

The following supporting information can be downloaded at: https://www.mdpi.com/article/10.3390/cancers14184463/s1, Figure S1: Effects of anti-PTK7 monoclonal antibodies (mAbs) on cytotoxicity of human umbilical vein endothelial cells (HUVECs); Figure S2: Original Western blot images for Figure 1B; Figure S3: Original Western blot images for Figure 9; Figure S4: Original Western blot images for Figure 10; Table S1: Primers used for polymerase chain reaction amplification of complementary DNAs (cDNAs) encoding human PTK7-Ig1-5-His, PTK7-Ig1-4.2-His, and PTK7-Ig1-3-His; Table S2: Primers used for *Dpn*I-mediated mutagenesis to generate deletion constructs expressing human PTK7-Ig1-4-His, PTK7-Ig2-4-His, and PTK7-Ig3-4-His.
