# Supplementary material for: Anti-PTK7 Monoclonal Antibodies Inhibit Angiogenesis by Suppressing PTK7 Function
Source: Cancers (Basel). 2022 Sep 14;14(18):4463. doi: 10.3390/cancers14184463 (PMC9496920; doi:10.3390/cancers14184463)
Supplement: Supplementary file 1 [file cancers-14-04463-s001.zip › cancers-1887877-supplementary.pdf]

Supplementary Materials

Anti-PTK7 Monoclonal Antibodies Inhibit Angiogenesis by Suppressing PTK7 Function

Si Won Oh, Won-Sik Shin, and Seung-Taek Lee*

Department of Biochemistry, College of Life Science and Biotechnology, Yonsei University, Seoul 03722, Korea
* Correspondence: stlee@yonsei.ac.kr; Tel.: +82-2-2123-2703

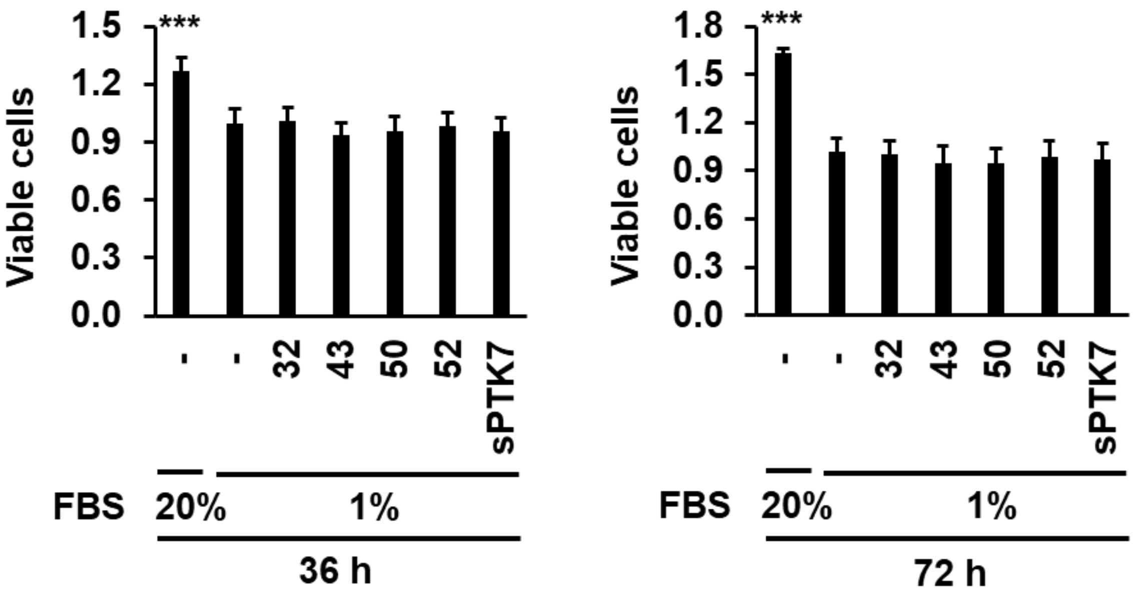

Figure S1. Effects of anti-PTK7 monoclonal antibodies (mAbs) on cytotoxicity of human umbilical vein endothelial cells (HUVECs)

HUVECs (4×10^3 cells/well) were seeded in gelatin-coated 96-well plates. After 24 h, the cells were incubated in M199 medium with 1% fetal bovine serum (FBS) for 36 and 72 h for cytotoxicity by starvation, in the presence of mAb-32, mAb-43, mAb-50, or mAb-52 (10 µg/mL) or soluble PTK7 (sPTK7; 4 µg/mL). The number of viable cells was measured using the 3-(4,5-dimethylthiazol-2-yl)-2,5-diphenyltetrazolium bromide assay. Each bar represents mean ± standard deviation (n = 6). *** $p < 0.001$ vs. the control incubated in 1% FBS medium. The viability percentage was not significantly different between the control (1% FBS) group and the PTK7 mAb-treated or the sPTK7-treated groups.

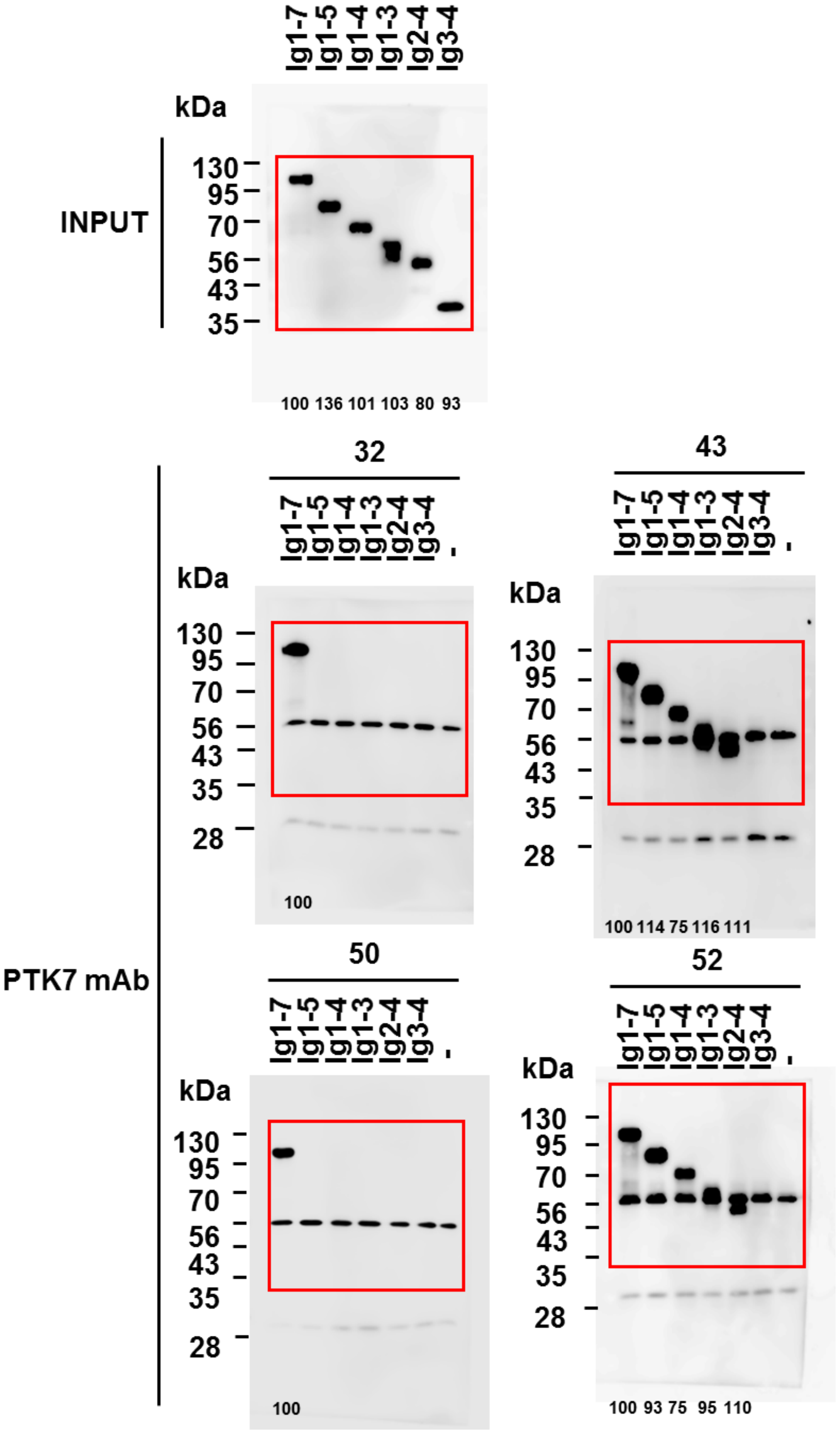

Figure S2. Original western blot images for Figure 1B

kDa: kilodalton

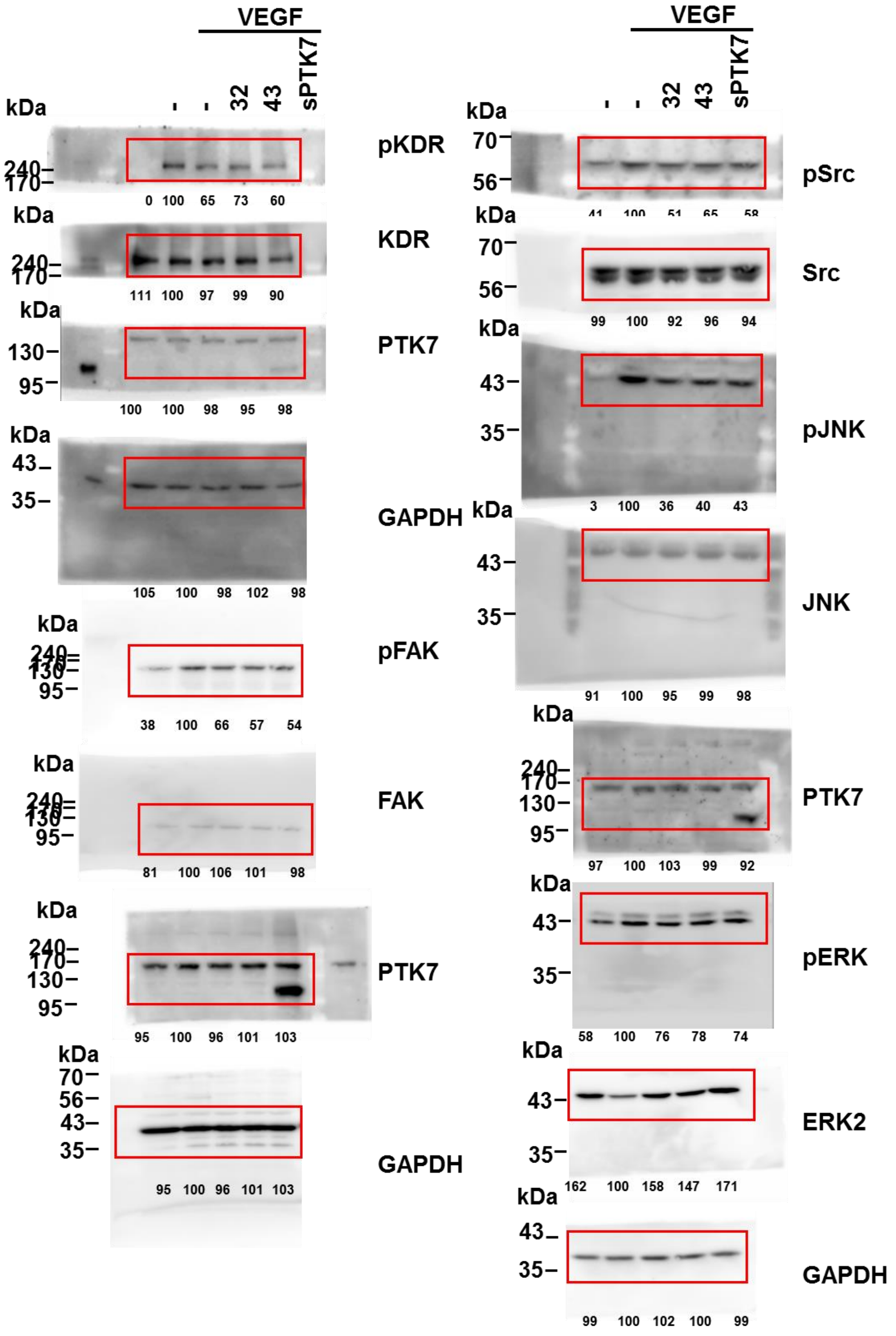

Figure S3. Original western blot images for Figure 9

kDa: kilodalton

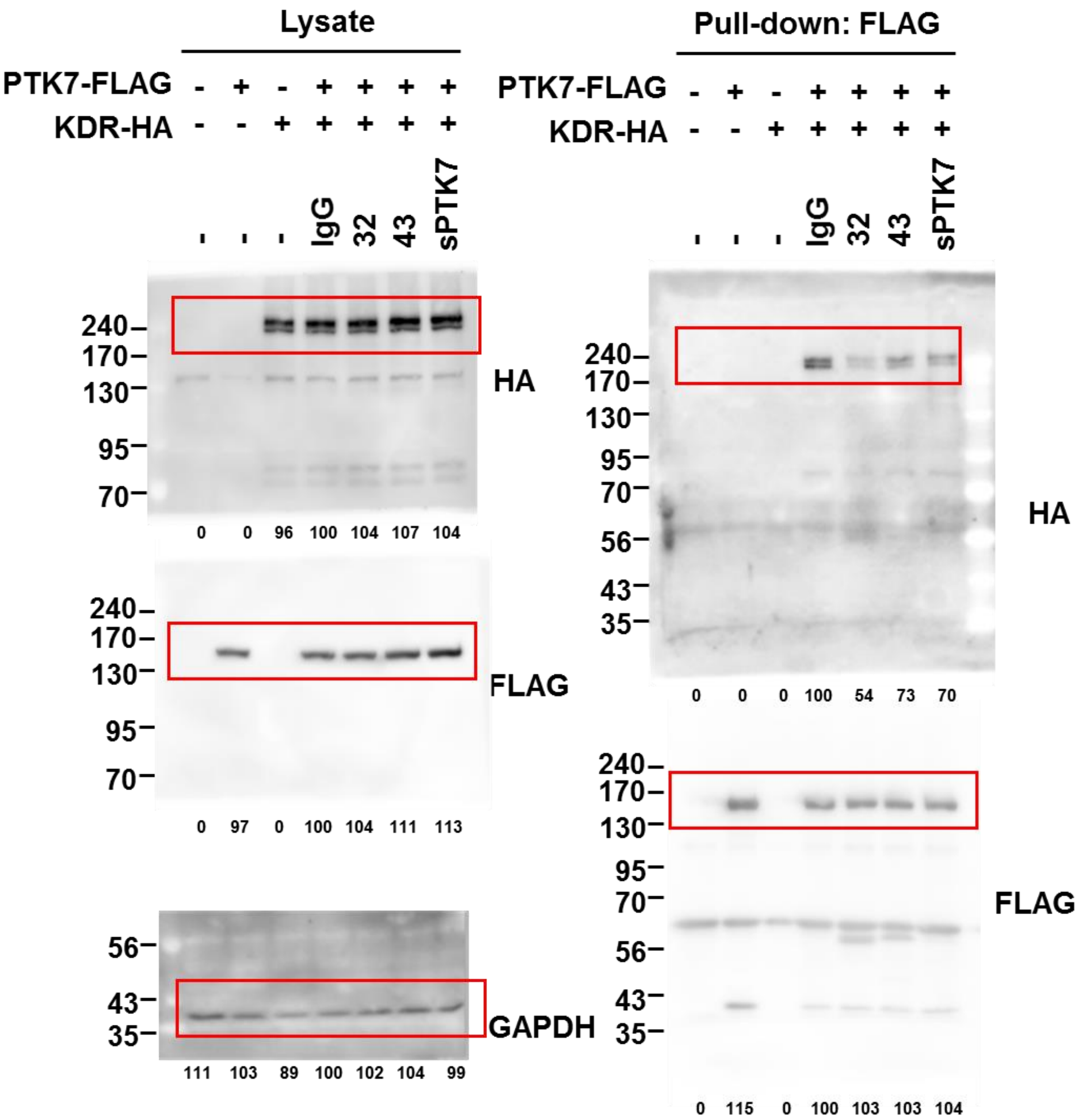

Figure S4. Original western blot images for Figure 10

kDa: kilodalton

Table S1. Primers used for polymerase chain reaction amplification of complementary DNAs (cDNAs) encoding human PTK7-Ig1-5-His, PTK7-Ig1-4.2-His, and PTK7-Ig1-3-His

Primer name[1]	Nucleotide sequence[2]	Nucleotide position[3]
Ig1-F	5′-TAATACGACTCACTATAGGG-3′	863-882 of MN996867
Ig5-His-R	5′-GC*TCTAGA***TCA**ATGATGATGATGATGATGGCCTGTGGCTGAACAGGG-3′	1729-1746 of U40271
Ig4.2-His-R	5′-GC*TCTAGA***TCA**ATGATGATGATGATGATGCTGGGTCAGGCAATCCAA-3′	1470-1453 of U40271
Ig3-His-R	5′-GC*TCTAGA***TCA**ATGATGATGATGATGATGAAGGCAGGTCACACGCTC-3′	1194-1177 of U40271

[1] F: forward primer and R: reverse primer

[2] Sequences derived from pcDNA3.1(+) and human PTK7 cDNA are shown in blue and red, respectively. Sequences corresponding to the *Xba*I restriction site (TCTAGA), stop codon (TGA), and His tag are shown in italics, bold, and underline formats, respectively.

[3] MN996867 and U40271 indicate GenBank accession numbers for pcDNA3.1(+) and human PTK7 cDNA, respectively.

Table S2. Primers used for *Dpn*I-mediated mutagenesis to generate deletion constructs expressing human PTK7-Ig1-4-His, PTK7-Ig2-4-His, and PTK7-Ig3-4-His

Primer name[1]	Nucleotide sequence[2]	Nucleotide position[3]
Ig1-4-His-F	5′-CATCACTGTGGCCCATCATCATCATCATCAT**TGA***TCTAGA*GGGCC-3′	1377-1389 of U40271 and 997-1001 of MN996867
Ig1-4-His-R	5′-GATGATGATGATGGGCCACAGTGATGTTGACATCCTGTCTC-3′	1389-1362 of U40271
Ig2-4-His-F	5′-GTCTTCATCAAGCAGTGGATTGAGGCAGGTCCTGTGGTCC-3′	259-273 and 529-553 of U40271
Ig2-4-His-R	5′-CCTGCCTCAATCCACTGCTTGATGAAGACAATGGCTGTCTGG-3′	542-529 and 273-246 of U40271
Ig3-4-His-F	5′-GTCTTCATCAAGCAGGATGAAAGCTTTGCCAGGGTGGTGC-3′	259-273 and 823-847 of U40271
Ig3-4-His-R	5′-GGCAAAGCTTTCATCCTGCTTGATGAAGACAATGGCTGTCTGG-3′	837-823 and 273-246 of U40271

[1] F: forward primer and R: reverse primer

[2] Sequences derived from human PTK7 cDNA and pcDNA3.1(+) are shown in red and blue, respectively. Sequences corresponding to the *Xba*I restriction site (TCTAGA), stop codon (TGA), and His-tag are shown in italics, bold, and underline formats, respectively.

[3] U40271 and MN996867 indicate GenBank accession numbers for human PTK7 cDNA and pcDNA3.1(+), respectively.
